# Proposed Ancestors of Phage Nucleic Acid Packaging Motors (and Cells)

**DOI:** 10.3390/v3071249

**Published:** 2011-07-20

**Authors:** Philip Serwer

**Affiliations:** Department of Biochemistry, The University of Texas Health Science Center, San Antonio, TX 78229, USA; E-Mail: serwer@uthscsa.edu; Tel.: +1-(210)-567-3765; Fax: +1-(210)-567-6595

**Keywords:** abiotic ancestors, bacteriophage structure, biological energy transduction, information-carrying polymers, thermal ratchet

## Abstract

I present a hypothesis that begins with the proposal that abiotic ancestors of phage RNA and DNA packaging systems (and cells) include mobile shells with an internal, molecule-transporting cavity. The foundations of this hypothesis include the conjecture that current nucleic acid packaging systems have imprints from abiotic ancestors. The abiotic shells (1) initially imbibe and later also bind and transport organic molecules, thereby providing a means for producing molecular interactions that are links in the chain of events that produces ancestors to the first molecules that are both information carrying and enzymatically active, and (2) are subsequently scaffolds on which proteins assemble to form ancestors common to both shells of viral capsids and cell membranes. Emergence of cells occurs via aggregation and merger of shells and internal contents. The hypothesis continues by using proposed imprints of abiotic and biotic ancestors to deduce an ancestral thermal ratchet-based DNA packaging motor that subsequently evolves to integrate a DNA packaging ATPase that provides a power stroke.

## 1. Introduction

Analysis of biotic motors (*i.e*., motors constructed with information from polymer coding) has focused on the question of whether or not a power stroke occurs. A power stroke implies movement of a load by reaction to parallel, ATP cleavage-driven motion of a region of the motor. An alternative is movement of a load by thermal motion of the load with ATP cleavage-dependent selection against thermal motion in the “wrong” direction (thermal or Brownian ratchet mechanism). More generally, biasing of a thermal ratchet can occur via non-specific forces, such as those generated by either electrical fields or pressure gradients. These alternatives have been considered for actin/myosin, kinesin/tubulin, dynein/tubulin, ribosome/peptide and phage DNA packaging motors [1–8]. Phage DNA packaging motors cause entry of a double-stranded DNA molecule (*i.e*., the load) into a cavity of the protein shell of the phage capsid. The shell is typically made of an icosahedrally symmetrical lattice of identical copies of one or, sometimes, more proteins.

**Figure 1:**
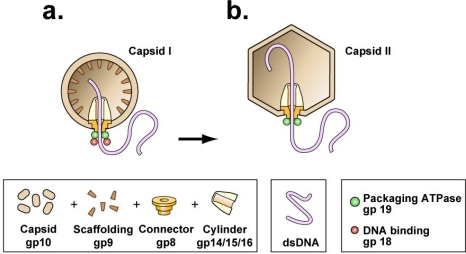
The initial stages of DNA packaging of the related phages, T3 and T7 (adapted from [18]). The legend at the bottom indicates the means of representation of both the DNA molecule and the various proteins. Proteins are named by gp, followed by the number of the encoding gene. The internal cylinder (gps 14–16) is a feature not present in some double-stranded DNA phages and its evolution is not discussed here. The DNA binding protein (gp18) is a general feature of connector-dependent phages, but its evolution is also not discussed here.

Recent data, obtained with phage ϕ29, indicate that phage DNA packaging motors have a cycle (to be called the type 1 cycle) that does have a power stroke [9]. The consensus opinion favors a power stroke delivered by a universally found DNA packaging ATPase [10–15]. Several ATPase molecules form a ring attached to a second ring called the portal ring or connector. The connector is attached to the shell at one of the shell’s axes of five-fold rotational symmetry, thereby creating a symmetry mismatch because the connector has twelve subunits (illustrated for the related phages, T3 and T7, in Figure 1) [10–15].

Alternative proposals have been made for the delivery of the power stroke. Delivery by the connector is favored by studies of mutants of the connector of phage SPP1 [16,17]. In addition, my explanation of data of all types produced the following proposal for the type one cycle: The DNA packaging ATPase ring acts as an ATP cleavage-driven, bind/release thermal ratchet that transfers energy to the connector; the connector delivers the power stroke [18]. As I have previously discussed [18], the proposed ATPase-to-connector energy transfer resembles energy transfer by ABC transporters. The energy transfer of ABC transporters is sometimes inter-domain and sometimes inter-protein [19–21]. Possibly, the ABC transporters evolved from phage DNA packaging motors.

The point has been well made that understanding of any biotic system requires consideration of its history, *i.e.*, the sequence of its ancestors, in addition to consideration of the system’s current biochemistry and biophysics [22,23]. A power stroke-based nucleic acid packaging motor is more complex and, therefore, presumably is the product of more adaptive steps than a thermal ratchet-based counterpart. Thus, one reasonably conjectures that each component of a power stroke-dependent biotic motor has an ancestor that was part of a thermal ratchet. In the case of DNA packaging motors, the following data suggest that connectors evolved before DNA packaging ATPases and, therefore, that a connector is part of a thermal ratchet-like ancestor that did not have the DNA packaging ATPase: (1) The shell is essential to DNA packaging and, therefore, must have been present for all ancestral DNA packaging. (2) The connector is embedded in the shell. (3) The DNA packaging ATPase is attached to the connector (reviews: [11–13,18,24]; see Figure 1).

Thus, I attempt here to deduce the characteristics of a thermal ratchet-based, DNA packaging ATPase-independent (but connector-dependent and high-energy compound-dependent) ancestor to the type 1, power stroke-dependent motor mentioned above. In so doing, I work with the constraint that the hypothesis be consistent with a reasonable proposal for ancestors of the thermal ratchet-based motor that are ancient enough to be abiotic. Thus, the results include new proposals concerning some general aspects of abiotic (sometimes called pre-biotic or pre-evolution) chemistry. The focus on nucleic acid packaging motors has the advantages of (1) the information already obtained for the present-day motors already studied (to be called classical motors) and (2) the potential information to be obtained, in the future, by detecting and characterizing additional present-day motors that are less evolved than the classical motors (pre-classical motors).

## 2. Nucleic Acid Packaging Motors

### 2.1. An Unexplained Aspect of DNA Packaging

Studies of sequence- and structure-based similarity support the assumption that classical phage DNA packaging motors have ∼1.6 billion year-old ancestors that have the components, and presumably the basic mechanism, of the classical motors. These studies include findings of sequence similarity for two of the three critical components of the DNA packaging motors of double-stranded DNA phages and also eukaryotic viruses. These components are (1) the outer shell, for which a variation of one of two basic folds (double β-barrel- and HK97-folds) is found in all studied double-stranded DNA viruses and sequence-based similarity is typically (but not always) detected [25–29], (2) the DNA packaging ATPase, for which sequence similarity is typically detected [24,30–35] and (3) the 12-fold symmetrical connector, which, thus far, has only been found for viruses with the HK97-type outer shell protein, including herpes viruses. Sequence similarity is typically not detected for connector proteins, although structural similarity is [17,36–38]. In addition, terminase is a core viral gene [39], *i.e.*, without known function for cells. Terminase is, therefore, not likely to have been transmitted horizontally. Thus, classical, connector-dependent DNA packaging motors existed before the prokaryote/eukaryote splits, *i.e.*, by about 1.6 billion years ago [40,41].

A classical, connector-dependent DNA packaging motor begins DNA packaging with the capsid in the procapsid state (capsid I for T3/T7; Figure 1a). For all studied, connector-dependent double-stranded DNA phages, except ϕ29, the procapsid expands during packaging to become more like the mature phage capsid (capsid II in Figure 1b) (reviewed in [11,42–44]). Although extensive studies have been conducted of the various aspects of DNA packaging (reviews: [11,18,41–43]), I will focus here on the dynamics of the outer shell, including procapsid expansion, because the role of these dynamics in DNA packaging has never been explained and is potentially explained by the hypothesis to be presented here.

The need for an explanation for shell flexibility has recently increased with our finding that a second shell expansion occurs during phage T3 DNA packaging in an infected cell (*in vivo*), after the expansion that occurs during the capsid I to capsid II transition, *i.e.*, after the procapsid to mature capsid transition [45]. This second expansion is a hyper-expansion in that the shell becomes larger than the mature phage shell. The evidence was derived from characterization of particles produced by the interruption of DNA packaging in a T3-infected cell *in vivo* [45]. Our interpretation was that (1) the T3 DNA packaging motor begins packaging with a connector/packaging ATPase-driven type 1 cycle that eventually undergoes an unreversed stall, in part because of the accidental packaging of non-DNA molecules, such as peptides and RNAs, and (2) this stall triggers the start of a second, contraction/hyper-expansion-based cycle (type 2 cycle). The contraction of the type 2 cycle is accompanied by increase in the permeability of the shell and binding of the DNA molecule by the connector so that accidentally packaged non-DNA molecules are expelled and the type 1 cycle re-started. The hyper-expansion of the type 2 cycle is accompanied by decrease in the permeability of the shell and release of the DNA molecule so that the DNA molecule continues packaging while entry of non-DNA molecules is inhibited (for further details, see [46]). Re-starting of a stalled type 1 cycle via mechanisms other than a type 2 cycle has been empirically detected directly by single-molecule analysis of *in vitro* phage ϕ29 DNA packaging [9,47] and by inference from the results of introducing either single-stranded breaks (nicks) or mismatches to a DNA duplex before phage T4 *in vitro* packaging [15,48].

### 2.2. Variations on a Theme: Packaging without a Connector

Packaging-associated shell expansion is also a feature of phage motors that package multiple single-stranded RNA segments. These single-stranded RNA segments are converted to double-stranded RNA segments inside the cavity of the capsid’s shell; the phages with these motors are called *Cystoviridae*. The *Cystoviridae* include ϕ6, ϕ8, ϕ12 and ϕ13 (reviews: [49,50]). The RNA packaging motors of these phages also have a packaging ATPase, but this ATPase functions without a connector. Nonetheless, as each genomic RNA molecule is packaged, its compartment within the shell expands, as determined by cryo-electron microscopy of phage ϕ6 [49,51].

In the case of the double-stranded DNA-containing *Tectiviridae*, another variation of packaging-associated expansion occurs. In this case, the protein shell of the capsid surrounds an inner shell of phage protein-enriched host membrane. This membrane forms the cavity in which DNA is packaged. Packaging is through a unique outer shell vertex and a packaging ATPase has been found. However, a connector has not been found [52]. Although expansion of the protein shell does not accompany DNA packaging, the DNA-enclosing membrane tracks the inner surface of the outer protein shell more closely in the mature virion than it does in the procapsid [53]. Thus, a DNA packaging-associated expansion of the DNA-enclosing cavity appears to occur in this case also.

When one considers only the biochemistry and biophysics, packaging-associated expansion of the nucleic acid-enclosing region of the viral capsid, which occurs for almost all known double-stranded DNA phages and the RNA-containing *Cystoviridae*, has no known selective advantage. Additional room could have evolved more simply with an original shell that had a larger radius. In fact, most hypotheses for packaging ATPase-based motors do not even include the shell expansion as part of the motor [10–15]. Thus, I have made the assumption that shell expansion occurs for reasons of history, not for reasons of history-independent biochemistry and biophysics. Indeed, shell expansion is simple enough to originate in an abiotic event.

Thus, in the present exercise, I use the shell expansion as a point of departure for a hypothesis that describes the nature of abiotic ancestors to modern viruses (and cells). The hypothesis includes a proposal for abiotic ancestors that occurred before the advent of polymer coding, which was perhaps 3.8–4.5 billion years ago [40]. Among the proposed ancestors, I also include a biotic DNA packaging motor with a thermal ratchet-based cycle. This motor is an ancestor to motors with a DNA packaging ATPase-, power stroke-based (type 1) cycle.

## 3. Objective and Assumptions

In the present communication, the objective is to deduce a plausible sequence of ancestors, beginning with abiotic ancestors and ending with a classical DNA packaging motor. Importantly, at least some aspects of a proposed ancestor sequence are expected to be testable via the isolation of phages from niches that favor pre-classical nucleic acid packaging motors. The theory has the potential to provide clues to where and how to find living versions of such anciently derived phages. Obviously, assumptions will have to be made because neither the abiotic ancestors nor the early biotic ancestors have been observed.

Thus, the above data are complemented here by the following plausible assumptions. First, I make the relatively generic assumption that the earliest ancestors are completely abiotic in character. That is to say, the earliest ancestors occur for reasons of chemistry/geology only and do not include a component of information-carrying polymers. This assumption excludes consideration of the arrival of information-carrying polymers from an extraterrestrial source, another planet, for example (exogenesis). If, nonetheless, exogenesis did occur, the abiotic ancestors proposed here are considered to occur at a location other than planet Earth. The onset of biotic ancestors for DNA packaging motors presumably intersects the onset of all biotic ancestors.

Second, I make the assumption that the earliest ancestors occur in regions of the Earth that have a relatively high concentration of molecules in general and of precursors for “biotic” molecules in particular. The more concentrated the precursors, the higher the probability that productive, but random and rare, events occur in succession, as required for the onset of biotic events. As discussed elsewhere, this region of the Earth is likely not to be at the surface (because of effects of solar radiation [54,55]) and not to be within a lake- or ocean-sized body of water (because of relatively low concentration) [40,56]. But, the exact location is not a component of the theory presented here; possibilities are discussed after presentation of the hypothesis.

Third, I make the assumption that all energy transduction of the earliest ancestors occurs via changes in chemical bonding that occur relatively frequently; changes in non-covalent bonding may be involved. This assumption is based on the notion that the most frequent changes in either covalent or non-covalent bonding are the most likely to generate, by chance, the non-information-dependent (abiotic), earliest ancestors.

Fourth, I make the assumption that the abiotic ancestors are involved in protective transport that is driven abiotically, *i.e.*, by geological forces and diffusion. The rationale for this assumption is that transport increases the probability of productive, although uncontrolled, abiotic changes by increasing the diversity of the compounds with which any given compound comes in contact. The protective aspect maximizes the potential for preserving relatively unstable molecules during transport. This assumption introduces the concept of transport-dependent chemical potential gradients, a concept that differs from the current consensus of chemical potential gradients that do not depend on transport (reviews: [40,56,57]).

The fourth assumption is indirectly justified by its explanation of two otherwise paradoxical observations. These two observations are near-universality of the genetic code and unique chirality of biotic molecules, for example, sugars, amino acids and nucleotides [55,58]. The proposed explanation for both begins with the fourth assumption applied to ancient biotic systems that were not yet motile, *i.e.*, the RNA cells discussed below. That is to say, even though not yet motile, the ancient biotic systems moved enough for content exchange sufficient to bring all of them in contact with each other. The explanation for the near-universality of the genetic code concludes with the highly plausible assumption that this exchange was essential to convergence of initially arising codes to an optimized code that is empirically known to be (almost) universal [58]. The explanation for unique chirality concludes with the following consequence of a gene pool broadly accessed via transport. Whatever the chirality was that initially became dominant by chance, it remained dominant because molecules with the opposite chirality were the products of synthesis via a smaller gene pool. Therefore, synthesis could not evolve as efficiently [55,56]. When combined with the second assumption, the fourth assumption implies that the first polymer-encoded information occurs with molecules that are at relatively high concentration and that are undergoing protective transport.

Fifth, I re-state the assumption that the classical nucleic acid packaging motors studied thus far retain enough characteristics of their abiotic ancestors so that one can use the classical motors as a starting point for deducing the characteristics of the abiotic ancestors. This fifth assumption is the most uncertain assumption, of course. It is rooted in the conclusions (discussed above) that (1) the components of today’s DNA packaging motors have ancient origin, (2) capsid expansion associated with nucleic acid packaging is an unexplained phenomenon that is potentially rooted in the ancient past, and (3) a thermal ratchet-based motor is likely to have been one of the earliest ancestors. In other words, the fifth assumption is that some aspects of classical nucleic acid packaging motors are imprints (sometimes also called relicts) from an ancient period (for the basic concept, see also [40,56,59]). In connecting classical DNA packaging motors with their ancestors, the current exercise attempts to explain observations that are not easily explained by geological time-independent considerations of biochemistry and biophysics only.

## 4. The Hypothesis

### 4.1. Approaching the Abiotic/Biotic Barrier with Shells as Carrier

**Figure 2:**
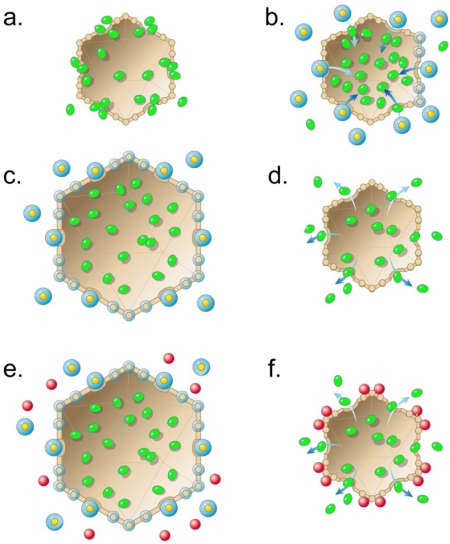
The first proposed abiotic ancestor and its transformations. Tan, abiotic shell-forming atom; yellow, high-energy compound; blue, water cage; green, organic molecules; red, product of reaction of the green organic molecules.

The hypothesis starts by proposing that, for both viruses and cells, the earliest abiotic ancestors are hollow, porous shells that are unanchored and, therefore, capable of being transported. These abiotic shells (1) are made of geologically generated compounds that happen, because of their chemistry and surrounding conditions, to have an internal cavity, (2) exist in a region of highly concentrated organic molecules, some of which are ancestors of biotic polymers, and (3) have open regions (pores) so that the internal cavity can be filled with one or more of the organic molecules. Figure 2a illustrates an icosahedral abiotic shell in the presence of organic molecules, the latter represented by solid, green ellipsoids. The pores of the abiotic shell are drawn as circular with the edge of some of the pores bound to organic molecules.

Although shells of this type appear not to have been observed, related shells have been observed for rare earth borides. The rare earth borides form crystals of interconnected octahedral and icosahedral shells of boron, with details dependent on the molar metal/boron ratio. For some of these compounds, a single rare earth metal atom is packaged at the center of a shell [60,61]. The rare earth metal/boron shells do not, however, have pores like those pictured in Figure 2a. The proposed abiotic shells are essential to crossing the barrier between abiotic and biotic events.

The proposed abiotic shells of Figure 2 are transport vehicles, although relatively inefficient. They move via either diffusion or geologically derived forces (from earthquakes, for example). When, by chance, an organic molecule diffuses through a shell’s pore without adhering to the edge of the pore, this molecule becomes packaged in the cavity of the shell. If subsequently either the pores are closed (a possible mechanism is described below) or binding to the shell interior occurs, then the packaged molecules are protected during transport, which eventually takes the organic molecules away from the site at which they normally exist and are relatively stable. A consequence of transport is that packaged organic molecules have an increased chance of exposure to both molecules of diverse chemistry and differences in chemical potential that are greater than experienced without transport. The organic molecules, therefore, also have an increased chance of being part of a chain of events that eventually includes formation of an information-carrying polymer. Thus far in the hypothesis, assumptions 1–4 have been incorporated.

The hypothesis incorporates assumption 5 by continuing with the following. Among the various abiotic, transporting shells, some are, by chance, part of an abiotic, chemical energy-driven system that makes the shells more transport active via closing of the pores. The chemical energy comes from the binding of relatively high-energy compounds (represented by relatively large spheres with yellow centers in Figure 2b) to the shell. This binding progressively closes each of the shell’s pores and, while doing so, causes (1) entry into the shell’s internal cavity of molecules that had been bound at the entrance of a pore, (2) progressive decrease in the permeability of the shell, and (3) increase in radius of the shell (Figure 2c), additional details of which are proposed in the next paragraph. In Figure 2, these changes are illustrated via the binding of the high-energy compound(s) to the edges of the pores, thereby displacing the previously bound molecules and sterically plugging the pores; a more allosteric mechanism is also within the boundary of the hypothesis. The increase in shell radius is the proposed origin of an imprint transmitted through subsequent abiotic and, later, biotic ancestors. This imprint is the source of the nucleic acid packaging-associated expansion of the shells of the capsids of both DNA and RNA phages.

I also propose here a mechanism for the increase in shell radius illustrated in Figure 2c. Whether or not correct in detail, this mechanism illustrates the feasibility of such an expansion. To increase shell radius, the abiotic shell-forming atoms of Figure 2 attract a caging compound to form a clathrate [62–64], an effect nucleated by binding of the high-energy compound. Based on current experience, the caging compound is most likely water [62–65] and the high-energy compound is either methane hydrate [64] or a hydrate of another abundant compound. The high-energy compound in Figures 2b,c is drawn with a blue ring around its yellow center to indicate the likelihood of it being a hydrate. The shell expands because of a cooperativity-of-hydration [66] induced increase in hydration of the shell-forming atoms, as illustrated by the addition of a blue ring to the atoms of the shell in Figure 2c.

In summary, the binding of the high-energy compound to the abiotic shells has the effect of pumping molecules into the shell and keeping them there. This pumping occurs via (1) displacement by the high-energy compound of molecules bound at a pore (as illustrated in Figures 2b,c) and (2) increase in volume that occurs before the permeability is reduced close to zero. Other mechanisms more allosteric than displacement are also possible. This packaging is the first step in enhancing transport.

The second step is increasing the retention of the packaged molecules during transport away from regions in which the packaged molecules are in relatively high concentration. This step occurs, by accident, via the relatively broad distribution in the environment of the high-energy compound. That is to say, the high-energy compound is relatively (but not completely) omnipresent and keeps the shells sealed while the packaged molecules are transported among regions of relatively low packaged molecule concentration (Figure 2c).

The enhanced transport eventually brings the shells to a region in which the high-energy compound has a relatively low concentration. When that happens, the high-energy compound dissociates from the shells, thereby causing contraction and permeability increase of the shell. The packaged molecules now diffuse into a new environment. Thus, the range of potential reactions is increased in relation to the range that existed without transport. Although inefficient in promoting chemical diversity by the standards of biotic events, the proposal here is that these energy-transducing, transporting shells provide the most efficient abiotic means for promoting synthesis of ancestor compounds in a sequence of events that ends with production of information-carrying polymers.

I emphasize that the ancestors in Figure 2 are not derived from a process of template-controlled reproduction. No biotic (*i.e*., no polymer coding-dependent) event exists at this point. Methane is the likely high-energy compound, given that methane is potentially generated throughout most of the geosphere via abiotically produced hydrogen (serpentinization reaction, for example) and the Fischer-Tropsch reaction (review: [67]). Suggestively, in boron carbides, carbon reacts with boron to connect icosahedra, rather than to be packaged within them [68].

One reasonably asks whether shell transitions like those of Figure 2 have a basis in chemistry that is already known, rather than the chemistry proposed here in rough outline. The answer is “not to my knowledge”. But, I make the point that known chemistry is also not even close to explaining the onset of biotic systems (review: [40]). Something is missing. I propose that the missing chemistry is the chemistry of abiotic shell hydration. The initial rationale for proposing expandable abiotic shells is discussed under the fifth assumption, above.

The energy-dependent, shell-based transport of Figure 2 occurs at the same time as other forms of abiotic transport. To have promoted the onset of information-carrying polymer formation, abiotic ancestor shells must have been a component of the environment for a time comparable to the time needed for the onset of information-carrying polymer appearance. Perhaps, then, these shells still exist.

### 4.2. Onset of Biotic Memory: Autocatalytic, Shell-Dependent Chemistry

The earliest memory-mimic (and precursor of memory) can occur only through an abiotic process that resembles a biotic process. To progress from the scenario of Figure 2, I adopt the previous proposal [69] that this abiotic process is autocatalysis. However, I propose autocatalysis that is more indirect than what was previously proposed. Specifically, I propose that some compounds produced by the process of Figures 2a–d (red spheres in Figure 2e) happen, by chance, to reversibly bind the shell and assist in shell contraction and release of shell contents (Figure 2f). The released shell contents include precursors of the product compound(s). Thus, the released shell contents accelerate the production of the product compound. That is to say, the process of Figures 2e and 2f is a form of indirect autocatalysis of the synthesis of the product compound. This indirect autocatalysis is most effective if the movement of shells from high- to low-energy environment is followed by return of significant numbers of them to the high-energy environment in a cyclic process.

**Figure 3:**
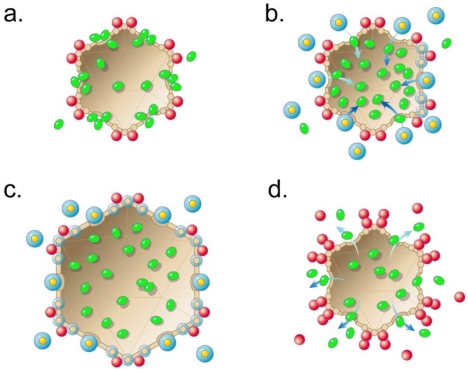
The transformations of Figure 2 altered by irreversible integration of product molecules in the abiotic ancestor shell. Tan, abiotic shell-forming atom; yellow, high-energy compound; blue, water cage; green, organic molecules; red, product of reaction of the green organic molecules.

The next stage in the succession of ancestors is like the previous stage (Figures 2e,f), except that the shell-binding molecules (red spheres) bind the shell irreversibly, rather than reversibly. The binding is strong enough so that the shell-binding product molecules remain with the shell (Figure 3a) while the shell packages a new collection of precursors with the assistance of a high energy compound(s) (Figures 3b,c) and returns to a place in which some of these product molecules have already been made (Figure 3d). This place is not necessarily the same as the place of the synthesis of the shell-associated product molecules, but the conditions are related.

After the shells return to the condition of Figure 3d, the autocatalytic process of shell content release becomes more efficient because of the binding of external product molecules to counterparts that are shell-bound. This dimerization-like event, illustrated by the red spheres in Figure 3d, increases the efficiency of the cyclic process of Figures 2 and 3. Thus far, no aspect of any of these processes is biotic, although the process in Figure 3 has become the most competitive for producing a reaction product.

**Figure 4:**
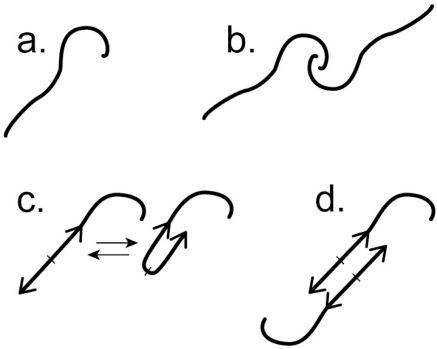
Palindromes and the intramolecular and intermolecular interactions of product molecules. (**a, b**) Earliest monomer and dimer; (**c, d**) Monomer and dimer of molecules with partial palindromic character.

I propose, however, that a biotic stage is approaching via dimerization of the reaction product compounds. A relatively featureless monomer of a reaction product compound (*i.e*., a compound represented by red spheres in Figure 3) is pictured in Figure 4a and its dimer in Figure 4b. Eventually, by accident, some of these compounds acquire a feature that is essential to further changes toward a biotic system. This feature is partial palindromic character, in the sense of a (self-complementary) nucleic acid palindrome. The partial palindromic character is illustrated in Figure 4c by two arrows, one inverted relative to the other. Although only partial, the palindromic character is sufficient to introduce some secondary and tertiary structure in the monomer, as illustrated at the right of Figure 4c. Importantly, the partial palindromic character is also sufficient to support dimerization (illustrated in Figure 4d). Possibly, but not necessarily, these molecules are an approximation of the shorter oligonucleotides of today. With the change of Figures 4c, d, the process of Figure 3, although abiotic, is on its way to autocatalytically generating ribozyme-like molecules with capacity for catalysis.

Eventually, with a large leap for which only limited details are proposed in the next section, the product molecule becomes either RNA or a compound that evolves to RNA during the biotic stage of ancestry. A RNA-based stage of biotic ancestry, with no DNA and no proteins, has previously been almost universally accepted [56,70–74] and is assumed here. In this “RNA world”, RNA has both coding and enzymatic function. However, the nature of the envelope that surrounds RNA cells remains a matter of conjecture. The primary basis for proposing the existence of an ancient RNA world is that RNA is the only molecule that is known to have both coding and enzymatic characteristics, as previously discussed [56,70–74].

Returning to the abiotic, pre-RNA world of Figure 3, I anticipate the next part of the hypothesis by indicating that I will propose that the product compound-containing shells from Figures 2 and 3 are ancestors of both cellular membranes and the shells of viral capsids. No distinction between viral capsids and cellular membranes yet exists. In addition, all reactions, thus far, occur outside of the shell.

### 4.3. Internalization: Onset of Polymer-Encoded Information

The hypothesis continues with the proposal that the ancestor progression-producing reactions eventually also occur in the internal cavity of the abiotic, transporting shells. This internalization of reactions has to occur in order for viruses and cells to arise. Specifically, as complex reaction products become more concentrated, the chance increases that the packaging and transport of Figures 2 and 3 occurs not only for relatively small precursors represented by the green ellipsoids, but also the more complex molecules that are the products of the event sequence of Figure 3, *i.e.*, some of the molecules represented by the red spheres. A result is that, during transport, chemical reactions occur among the smaller molecules and their more complex reaction products, all packaged in the abiotic, decorated shells in Figure 3. Otherwise, the sequence of events is that of Figure 3.

In the following ways, internalization of reactions accelerates the ancestor progression in the direction of biotic ancestors. First of all, internalization sometimes concentrates molecules, which causes an increase in reaction speed and range. Increase in reaction speed and range decreases the time for abiotic reactions to produce a new ancestor. Second of all, solution conditions are determined in part by the enclosing shell, which accelerates progress in those shells that provide an environment that reduces hydrolysis, the primary barrier to the production of an encoding polymer of RNA, for example (review: [70]). Finally, the presence of a high surface-to-volume ratio makes more efficient the positive effects of surface chemistry, such as surface adsorption that stabilizes phosphodiester bonds [70,74]. Potentially, the high packaged molecule concentration and the modified environment exert some effects via reduced molecule hydration [70]. Reduced hydration occurs dramatically (factor of ∼3) for DNA packaged in classical double-stranded DNA phages [75]. Among the reactions eventually enhanced are polymerization reactions (possibly ribozyme-catalyzed ligation reactions) that produce the initial information-carrying polymers. A previous proposal postulates that these effects occur, in part, via freezing of the solution [70].

As time progresses, the packaged, quasi-palindromic molecules become increasingly more active in catalysis. Again, the basis for this change is change of the palindromic nature of these molecules, which gives them the secondary and tertiary structure needed. Progression accelerates again when the packaged ancestor molecules have catalytic activity that is expressed while packaged. Nonetheless, the ancestor progression is still abiotic in character, although conditions now exist to produce a molecule that not only has enzymatic activity, but also enzymatically duplicates by initially serving as a template for producing a complement to itself. Again, without proposing further details, I make the leap to RNA cells (*i.e*., cells with components dependent on both RNA catalysis and RNA-encoded information) in the next section.

### 4.4. Abiotic Shells and RNA Cells

The envelope of the universally accepted RNA cells has a composition that is a topic of conjecture. RNA cells preceded DNA-based cells and presumably had the handicaps of (1) no cellular biotic membrane evolved for optimization of uptake and release of compounds, (2) enzymatic activity provided only by ribozymes. and (3) information limited by the relative instability and, therefore, short length of RNA molecules. To give the RNA cell a more concrete basis, the proposal has previously been made that, initially, the envelope of RNA cells is abiotic in character. Possibilities include FeS walls that currently exist in hydrothermally formed iron sulfide chimneys under the oceans [40,56,57,76].

Here, I propose adjustment of this idea by the following continuation of the proposed sequence of ancestors, above. The envelope of the simplest RNA cells is a composite of RNA and abiotic shell. This composite is schematically (not literally) illustrated in Figure 3d, assuming that the ancestor progression reaches product molecules (red spheres and some of the packaged molecules in Figure 3d) that are RNAs.

In addition, RNA cells become more complex when aggregation and, then, merger occur among the simpler RNA cells. Merger causes mixing of packaged RNAs and other molecules, via opening of pores. Merger also causes mixing of RNAs that are integrated in the shell. I note that these RNA cells are still obtaining RNA precursors by the relatively inefficient process of Figure 3, although the shell-associated RNA has changed biotically (*i.e*., via evolution) so that those RNA molecules that provide a selective advantage are selectively retained when pores open. The metabolism, including templated RNA replication, occurs in the cavity of the shell. The retention of vital components during exchange with the environment is a characteristic that forms part of the foundation for further evolution.

Although polymer-encoded information and polymer-catalysis are both already part of what is now a biotic system, I note that capacity for transmission of the RNA-encoded information is limited by the absence of a shell that replicates along with the RNA and assists the intake of RNA precursors. That is to say, RNA replication occurs within a shell with no classical method for vertical transmission. Instead, vertical transmission occurs via recruitment of new abiotic shells by RNA molecules that have been synthesized in other shells and have been either extruded into the environment or transmitted via contact. In some cases, this recruitment involves displacing RNA molecules previously present. Thus, along with catalysis and polymer-encoded information, competition is also part of the RNA world at this stage.

In anticipation of the eventual production of protein-synthesizing cells, one imagines (in an embodiment simplified for illustration) that a RNA cell with a packaged, incipient precursor to tRNA merges with two other RNA cells, one with an incipient precursor to rRNA and the other to mRNA. This merger is an accident and is not guided by complementarity of the shell-incorporated RNA molecules. The result of the merger is an RNA cell that is a potential ancestor of a cell that has protein synthesizing capacity. As hazy as the details are, this idea does produce the concrete prediction that the first mRNA has palindromic character. Other mergers produce precursors of some viruses, although other viruses have single-shell ancestors.

Viruses and cells diverge from each other in the RNA world. A virus is differentiated from a cell in that the RNA incorporated in a virus shell has complementarity to some, but not all, RNA of a more complex RNA cell. That is to say, the virus has RNA molecules associated with at least one, but not all, of the shells that merged to form the RNA cell. Thus, the virus recognizes the RNA cell via complementarity of shell-incorporated RNAs.

The result is templated merger of the shells and their contents with outcome varying between two extremes. The first extreme is that the virus RNA out competes the other RNAs of the RNA cell and basically converts the RNA cell to a virus. This corresponds roughly to the classical infection with a lytic phage. The second extreme is that the virus provides a function that assists the RNA cell in becoming more efficient and competitive in propagating itself, still by the non-classical process described above. This outcome corresponds roughly to the classical infection with a temperate phage, but with more impact on the cell than is usually attributed to classical infection with a temperate phage. An infection of this type is basically a merger of the type that created RNA cells.

### 4.5. Proteins, Cells and Viral Shells

The hypothesis continues by proposing that, as a result of the various shell mergers and the reactions promoted by them, RNA cells acquire protein-synthesizing capacity and a biotic envelope. After first acquiring protein-synthesizing capacity, cells progressively substitute protein and other macromolecules for abiotic and RNA components of the shell until a protein-containing, completely biotic shell (cell membrane) evolves. Details are not proposed here. Viruses co-evolve a biotic shell because viruses are selected for efficiency of infecting whatever cells exist. Some details are discussed in the next section. Before the cell membrane replaces the abiotic component of the shell of RNA cells, exchange with the environment occurs by the process of Figures 2 and 3, which is inefficient compared to classical biotic processes.

Empirically, the existence of some RNA viruses that are segmented (*Cystoviridae*) does provide some support for this scenario. Specifically, the genomic segmentation of the *Cystoviridae* is accompanied by capsid segmentation to the extent that the various compartments of the capsid expand independently as their genome is packaged (discussed above). The capsid segmentation suggests an ancient merger of shells to form a single virus particle. The single virus particle retains the advantages of merger by converting merged shells to a single partitioned capsid and packaging multiple RNA molecules.

In addition, merger via either RNA-directed or random aggregation is likely to be endemic for both cells and viruses in the RNA world, for the following reason. Information in the RNA world is almost certainly not either transmitted or used with precision [56,57,72]. Therefore, redundancy is of selective advantage in the generation of biotic memory. The simplest means to achieve redundancy is aggregation. Thus, a test of the ideas presented here is possibly achievable by screening for short RNA-containing, aggregating viruses in the environment. Potentially, a comparatively unevolved descendent of the original merger-prone (*i.e*., aggregating) viruses still exists today and retains the imprint of compulsory aggregation. In fact, when we used techniques appropriate for isolation/propagation of aggregating phages [77], we found short RNA-containing phages that propagate in biofilm-like aggregates [78]. Genomic characterization of these latter phages, not yet performed, is potentially of interest for testing the ideas of this section and might be of additional interest because HTLV-1 (and possibly other eukaryotic RNA viruses) also propagates in biofilm-like aggregates [79].

Another apparent imprint from RNA world viruses is the following characteristic of *Cystoviridae*. As discussed above, *Cystoviridae* convert each of several packaged single-stranded RNAs to double-stranded RNAs while the RNAs are in the packaged state. This conversion might be considered surprising, if one does not consider the ancestry of viruses. However, the proposal made here is that this ancestry includes RNA world viruses that had (limited) capacity for ribozyme-dependent metabolism. Therefore, the retention of enzymatic capacity is understood as an imprint that survived the replacement of ribozymes with protein enzymes.

Parenthetically, I note that, even if the total number of phage particles in aggregates is high enough to detect by almost any procedure, the physical properties of phage aggregates make them difficult-to-impossible to detect by conventional procedures. The reason is that the aggregates are either lost during the removal of cells from a preparation or have properties so different from those of conventional single virus particles that they are not distinguished as virus-like. Biofilm-like phage aggregates have been propagated and detected via needle transfer of particles in zones of bacterial clearing in dilute (<0.1%) agarose gels. The constituent phages sometimes aggregate to the extent that they do not form single phage-originated plaques [77,78].

### 4.6. Protein After RNA: The Virus of Today

The hypothesis continues by proposing that the following occur as RNA cells acquire the capacity for synthesizing peptides via an RNA template: (1) substitution of peptides for RNA in the shells of Figure 3, (2) selective retention of the most competitive RNAs, located primarily in the shell’s cavity and (3) transfer to shell-associated peptides of the autocatalytic release-triggering function of Figure 3d. That is to say, the red spheres in Figure 3 become peptides, rather than RNAs. This change results in a more dramatic divergence of cells and viruses. As peptides replace RNAs in the abiotic shells, peptide dimers eventually provide the signal to release internal contents during either merger of shells or the process of Figure 3. However, the process of Figure 3 becomes progressively less significant as cells acquire protein/lipid membranes and the capacity for concentrating and digesting nutrients, as well as conducting metabolism and synthesis. I do not propose here any details for the sequence of ancestors of biotic cellular membranes and walls (see [80] for proposals in this area).

The hypothesis again continues by proposing that formation of these peptide dimers begins a process that continues to more extensive multimerization of peptides. In the case of both cells and viruses, peptide multimerization eventually produces a composite shell with protein molecules, and possibly some residual RNA molecules, in an array that physically is missing the abiotic shell component, but informationally is imprinted with the shape and symmetry of the abiotic shell. Subsequent divergent evolution produces (1) an all-protein viral shell, *i.e.*, what we now know as the shell of a viral capsid and (2) a cellular membrane that forms the envelope of the first completely biotic cells. The ancestor branch leading to completely biotic cells does not retain the original abiotic shell symmetry, but the ancestor branch leading to modern viruses does.

At least in part, the reason for this difference is that the most immediate RNA world ancestors of completely biotic cells are really each a product of the fusion of several shells. In contrast, the most immediate RNA world ancestors of completely biotic viruses are either the products of no fusions or the product of relatively few fusions, thereby reducing divergence from the symmetry of the original abiotic shells. In addition, virus infection becomes less contributory and more parasitic as cells evolve more capacity for synthesis and metabolism and are less dependent on outside input. Thus, viruses are not as selected as cells for divergence from the original symmetry. Nonetheless, even today, viruses still contribute at the genomic level and cannot be considered purely parasitic [81–84].

The ancestor succession proposed in the previous paragraph, although obviously missing many details, does have some empirical support. First, this succession predicts that the original scaffolding for viral shells is an abiotic shell that is at least sometimes symmetrical, based, in general, on the tendency for inorganic ions to form symmetrical structures [65] and, in particular, on the symmetry of the rare earth-boride shells [60,61]. Thus, this pathway also explains the otherwise puzzling observation that some double-stranded DNA viruses, including *Papillomaviridae*, SV40 and *Polyomaviridae*, have an icosahedral arrangement of pentamers. The all-pentamer aspect implies that local symmetry does not exist even though global symmetry does [85–87]. That is to say, by the hypothesis presented here, the global icosahedral symmetry of all-pentamer viral shells is an imprint of the original, abiotic shell, *i.e.*, a reflection of history, not a reflection of the biophysically most efficient mechanism of assembly given present circumstances.

In support, some capsids of double-stranded DNA *archaeal* viruses do not have either icosahedral or comparable symmetry and include spindle-shaped, bottle-shaped and droplet-shaped capsids [88–90]. The shapes of these archaeal virus shells are, by the hypothesis presented here, imprints of the shapes of other, ancient, abiotic structures, not yet identified. In the case of double-stranded DNA phages, the proposed abiotic scaffold leaves its imprint with the assistance of a phage-encoded protein scaffold usually, but not always [91], separate from the shell protein [91–94]. The protein scaffold for T3/T7 is formed by gp9 (Figure 1).

I note, however, that the proposed ancient divergence of cells and viruses does not preclude the subsequent reductive evolution, assisted by horizontal gene transfer, of some cells to viruses. Such reductive evolution could begin, for example, by parasitism of one cell by another and could continue by the progressive loss of unnecessary functions by the parasite [95]. This concept intersects the additional concept that a virus should be considered to be what we now consider to be a virus-infected cell [95,96]. Nonetheless, I retain the more traditional definition here. I do this, in part, because viruses are not always pure parasites, especially when viewed from the perspective of either the RNA world or the current microbial communities that undergo virus-dependent horizontal gene transfer. Thus, accretive evolution is also likely to have occurred, especially for some of the longer genome classical phages [81].

In conclusion, the concept of a common ancestor to viruses and cells has itself an ancestor in the proposal that viruses are the building blocks of cells. This latter proposal was made before the onset of modern molecular biology (reviews: [74,97]). Specifically, d’Herelle’s proposal that bacterial cells are made from phage building blocks [97,98], while nonsensical from the perspective of present biochemistry and biophysics, is far from nonsensical from a history-based perspective such as the perspective presented here.

### 4.7. From RNA to DNA

The introduction of DNA genomes is essential to increasing the coding capacity of cells, because of the instability of RNA, as discussed above. The hypothesis has been previously been presented that the transition to DNA genomes begins as a viral defense against host ribozymes that degrade RNA viral genomes. The viral use of a DNA genome is, by this hypothesis, then transferred to cells [71,99]. The hypothesis presented here (above) provides a basis for the presence of RNA viruses in the RNA world. Except for that, I propose nothing beyond the previous hypothesis [71,99], a hypothesis that I accept as probably correct.

Given the potential for increased information storage within a DNA (rather than RNA) genome, realization of that potential evolves for viruses, in part, by increasing the density of DNA packaged in the cavity of a viral shell. This evolution occurs by selection for the following two changes: (1) elimination or reduction of the amount of RNA and other non-DNA molecules in the cavity of the viral shell and (2) developing of both a mechanism and an energy source for condensing a DNA molecule to a higher density. The first change requires, in turn, a gatekeeper that selectively allows entry of DNA and restricts entry of other molecules.

**Figure 5:**
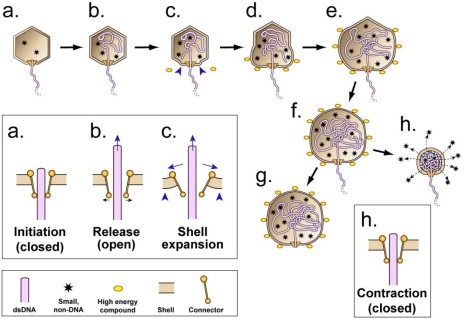
A proposed, pre-classical DNA packaging motor that has only connector and outer shell proteins. The legend at the bottom indicates the representation of DNA molecule, high-energy compound, packaged non-DNA molecule and capsid proteins. The insets (enclosed by rectangles) show the connector region of the corresponding capsid at higher magnification.

Thus, the hypothesis continues by proposing that selection for increased information carrying capacity produces the connector by selection for (and evolution of) a gatekeeper. This occurs before evolution of the DNA packaging ATPase. The connector recognizes and binds the end of a double-stranded DNA molecule (Figure 5a). Recognition of a DNA end also occurs in classical double-stranded DNA packaging motors. In the latter case, specificity in producing a packaging-enabling end is provided by process of cleavage of the genome from a concatemer, as found via studies of phages λ [100–102] and T3/T7 [103,104]. I propose that this feature of classical motors is an imprint from ancestral motors that have a connector, but not a DNA packaging ATPase.

The following observation is support (not proof) for evolution of the connector protein after the shell protein, even though sequence similarity is more readily observed for the shell protein [24–29] than it is for the connector [16,17,36]. The shell protein, assisted by its scaffolding protein, self-assembles *in vivo* to form a procapsid shell in the absence of the connector. This observation was originally made for phages T7 [105,106] and P22 [107]. Nonetheless, the T7 connector does increase the efficiency of assembly of shells by reducing mis-assembly [106]. Parenthetically, the *in vivo* studies of [105–107] are difficult to complement with reliable *in vitro* studies. The reason is that nucleation of procapsid assembly is likely to be at least a fifth order reaction, which requires the conditions of *in vitro* studies (shell and scaffolding protein concentrations and volume excluded by other molecules, for example) to be much closer to *in vivo* conditions than is likely to be achieved for an *in vitro* system without impractically large effort.

Figure 5a with inset illustrates the proposed first DNA packaging intermediate for each of several biotic ancestors produced by evolutionary selection for packaging that occurs increasingly more rapidly and to higher density. The resultant evolution begins with DNA packaging driven by a single shell expansion, the capacity for which begins with the cycles of Figures 2 and 3 and is transmitted as an imprint through RNA world ancestors that use the expansion for obtaining metabolites and for packaging RNA. In outline, the following are the specifics for the first one of these ancestors. Shell expansion is driven by binding of a high-energy compound, represented by yellow ovals with an orange rim in Figure 5; proposed details are in the next paragraph. The binding of the high-energy compound is accompanied by shell permeability decrease, as in Figures 2 and 3. The high-energy compound is possibly, but not necessarily, ATP. The permeability decrease is possibly, but not necessarily, the consequence of the plugging of pores by the high-energy compound.

In Figure 5, the process of high-energy compound-driven shell expansion is progressive. This expansion starts via opening of the connector (Figure 5b, inset), which (1) releases the DNA molecule for packaging and (2) causes a limited shell expansion, indicated by arrowheads in Figure 5c and inset. The limited expansion exposes high-energy compound binding sites on the shell; the high-energy compound binds to these limited sites. This binding of the high-energy compound causes a further expansion (Figure 5d), which continues with a wave of high-energy compound binding, interspersed with expansion/permeability decreasing, that spreads across the entire shell (Figures 5e,f).

The process of Figures 5a–f ends with packaging of the DNA molecule that had been bound at the connector; any external DNA is subsequently digested (Figure 5g). A source of the driving force for packaging is an osmotic pressure gradient across the shell; the pressure is lowest in the shell’s cavity. This osmotic pressure gradient is produced by the shell expansion, coupled with impermeability of the shell to molecules in the cytoplasm. Assuming intracellular osmotic pressure comparable to that in current cells, the density of the completely packaged DNA molecule is no higher than 30% of the density of DNA packaged by classical DNA packaging motors [18]. The density is likely to be lower because of inefficiencies that result in incomplete exclusion of non-DNA molecules from the cavity of the shell, for example. These latter molecules reduce the magnitude of the osmotic pressure gradient. The stars in Figure 5 represent the non-DNA molecules.

The DNA molecule will be injected into cells by a relatively inefficient mechanism related to the mechanism of expulsion of packaged molecules in Figure 3. No connector-attached injection organelle (tail) has yet evolved. The mechanism of injection includes removal of the high-energy compound from the shell, which now occurs enzymatically when the virus collides with a cell. That is to say, the hypothesis makes the following predictions: (1) A pre-classical virus of this type will have a high-energy compound, possibly ATP, attached to its shell. (2) This virus will also have a DNA packing density lower than the DNA packing density of classical phages by a factor of at least 3. (3) The cells infected by this virus will have envelope-associated enzymes that cleave high-energy compounds. The cells have these enzymes because they also have the expansion/contraction imprint and this imprint has been retained by evolutionary selection because it has a function. I do not propose specifics for this function.

After appearance of the motor of Figures 5a–g, a more advanced motor evolves. The more advanced motor packages more DNA because this motor undergoes more than one expansion and works as a cycling thermal ratchet. No additional phage-encoded proteins are necessary. I will propose the details for this more evolved motor after adding some perspective on the mechanism of Figures 5a–f by briefly discussing viruses that are different from those used for the current hypothesis.

### 4.8. Phages with a Shell That Does not Swell

These different viruses include the single-stranded RNA viruses that assemble without a protein scaffold and with assistance from the RNA genome in regulating subunit conformation. Included are several plant and insect viruses, as well as phages such as MS-2 and R17 (reviews: [108–110]). Although these single-stranded RNA viruses have the ancestry of Figures 2–4 in the hypothesis proposed here, the imprint from this ancestry has not remained to the extent that it has in the double-stranded DNA phages. The reason is that single-stranded RNA viruses have greater capacity to evolve away from the original mode of assembly. This capacity arises from the capacity of single-stranded RNA to undergo palindrome-like, intramolecular base pairing that produces secondary/tertiary structure in single-stranded RNA molecules at a level that cannot be achieved by a double-stranded nucleic acid. Thus, single-stranded RNA molecules can adapt structurally during co-evolution with the protein shell. Double-stranded nucleic acids cannot.

Thus, a spin-off of the basic hypothesis is that RNA/protein co-evolution produces an alternative mode of assembly for some single-stranded RNA viruses. In this alternative mode, single-stranded RNA forms a hydrogen bond-rich tertiary structure that serves as either a scaffold or a chaperonin for the subsequent assembly of protein subunits to form shells (to be called RNA-scaffolding viruses). That is to say, the RNA-scaffolding viruses have capsids with shells that are more diverged from the original abiotic shells than the shells of viruses that use protein scaffolds.

Phages with single-stranded DNA genomes have yet another packaging system. These phages have relatively few representatives among isolated viruses [111], the first and most studied of which is phage ϕX174. These phages also package DNA in a pre-assembled procapsid. This procapsid has scaffolds both internal to and external to the shell of the mature phage particle. The scaffolds are lost during DNA packaging. But, no expansion of the capsid occurs [94,112–114] and no connector exists. The motor function for packaging is apparently derived from DNA replication [114]. I propose that, like the RNA-scaffolding viruses, the packaging mechanism for phage ϕX174 and its relatives evolved relatively recently and has lost some of the imprint of the ancestors from Figures 2–4. In this case, more extensive divergence is again the consequence of the single-stranded character of the mature genome. But, in contrast to the RNA-scaffolding viruses, the basis of the more extensive divergence is the fact that a complementary strand is not produced during the stage of DNA replication in which the single-stranded DNA genome is packaged. Nonetheless, the following evidence exists for imprinting from ancestors common to the double-stranded DNA phages. The external scaffold has global, but not local, icosahedral symmetry, in analogy with the all-pentamer shells [94,112–114].

Other single-stranded DNA phages have a packaged DNA molecule folded on itself inside of a filamentous protein capsid (reviews: [113,115]). I do not attempt to incorporate these phages into the scheme presented here, because of both the single-stranded genome and the difference in shape and basic architecture.

### 4.9. DNA Packaging by Thermal Ratcheting

To achieve the high DNA packing density of classical double-stranded DNA phages (about 50% of the volume of the capsid’s cavity is occupied by the packaged DNA molecule [75,116]), evolutionary selection to increase DNA packing density must occur after the appearance of the earliest biotic ancestors. The proposal made here is that the virus of Figures 5a–g subsequently evolves to produce additional intermediates in a response to selection for increased information storage capacity and increased speed and efficiency of DNA packaging. This process ends with a chemical energy-dependent, bind/release thermal ratchet that pumps accidentally packaged molecules out of the cavity of the capsid’s shell. The chemical energy is used to drive shell expansion, as for the ancestors illustrated in Figures 2 and 3. The connector is used for bind/release, via the following cycle.

(1) As proposed for the ancestor virus of Figures 5a–g, the connector initiates packaging by undergoing expansion of its axial channel after the DNA molecule binds. This expansion allows entry of the DNA molecule into the cavity of the shell, while nucleating the force-producing, high-energy compound-dependent permeability decrease and expansion of the protein shell, as described above for the ancestor virus (Figures 5b–g). The energy for the shell changes is derived from binding of the high-energy compound to the shell. Eventually, the combination of both accidentally packaged smaller molecules (represented by stars in Figure 5) and inter-DNA segment repulsion generates enough opposing force to stop DNA entry (Figure 5f), if the packaging process goes no further.

(2) At this point, the more evolved virus does go further because further evolution has produced signaling capacity that is activated by slowing/stalling of packaging and causes two co-evolving changes in the response to stalling at the point of Figure 5f. The first change is removal of the high-energy compound from the shell. This change triggers contraction and permeability increase (Figure 5h) that cause expulsion of some of the non-DNA molecules (stars in Figure 5h). This expulsion makes possible the packaging of additional DNA. The second change is contraction-driven reversion of the connector of Figure 5h to the closed state of Figure 5a. The closing of the connector prevents the DNA molecule from being expelled from the shell’s cavity along with the non-DNA molecules. For efficiency of pumping non-DNA molecules out of the cavity of the shell, the contraction is likely to be driven to a radius smaller than the mature shell radius, as illustrated in Figure 5h; the energy for doing this is stored in the shell during the hyper-expansion.

In the transition illustrated by Figure 5f and Figure 5h, the high-energy compound is enzymatically removed from the shell, an event initiated by the lowering of the Gibbs free energy of the bound high-energy compound. This lowering of free energy occurs in a see-saw-like transfer of energy to the shell, initially to drive the expansion of Figures 5c. The proposed source of the enzymatic activity is the shell protein, because the proposed virus only has two consistently available proteins at this point and the connector is remote from the site of binding of the high-energy compound. That is to say, part of the hypothesis is the proposal that both the envelopes (membranes) of primitive cells and the protein shells of viruses have proteins with high-energy compound cleaving activity.

After the contraction, the shell spontaneously returns to its ground state, *i.e.*, the shell conformation of Figure 5b. This is the signal for initiation of another cycle; the next cycle is not shown in Figure 5. Cycles recur until the connector undergoes a change that causes it to (1) no longer initiate an additional cycle and (2) expose the external DNA segment to cleavage. The cause of this change is increase in the density of the packaged DNA. This role of the connector still exists in at least classical phages P22 [117] and SPP1 [118].

The cycle of Figure 5 is missing some aspects of classical DNA packaging motors. The missing aspects include the DNA packaging ATPase, as well as a precise means for terminating packaging and injecting the genome into a cell. These aspects evolve later with the motor of Figures 5a–f, h as an ancestor. The original high-energy compound might be, but is not necessarily, ATP. ATP is the source of energy for the DNA packaging of classical motors (reviews: [11,12]).

The evolution toward post-expansion shell contraction must occur with co-evolution of contraction-associated closing of the connector. Although steric connector closing (*i.e*., clamping of the DNA molecule) is the simplest type of connector closing, binding of the DNA molecule works as well. In support of an anti-DNA expulsion role of the connector during packaging, an anti-DNA expulsion role of the connector is known to exist in classical phages after DNA packaging is completed (phage P22: [119]; phage SPP1: [118]).

Finally, I emphasize that viruses with this pre-classical motor do not use a DNA packaging ATPase. I repeat that the basis for proposing the cycle of Figure 5 has two components, the known characteristics of classical DNA packaging motors and a plausible sequence of ancestors.

### 4.10. The Connector and a Packaging ATPase-Dependent Successor

The hypothesis concludes by proposing that the DNA packaging ATPase initially evolves to make the thermal ratchet-based motor of Figure 5 more efficient. Specifically, the earliest ancestors of the DNA packaging ATPase evolve to more efficiently perform the bind-release function of the connector. The reason for this proposal is that bind-release is a known function of classical DNA packaging ATPases [9,14], as reviewed in [18]. That is to say, the ancestral packaging ATPase genes are specialized in relation to the connector of Figure 5 and possibly are descendents of ancient connector genes.

The adoption of a second, specialized protein is selected because of the following two advantages: reduction of the effects of thermal noise on the signaling of the motor of Figures 5a–f,h [18] and increase in the rate of evolution via division of labor. Specifically, the sensing functions of the connector now evolve independently of selection for a DNA binding function because the ATPase now executes the DNA binding function. Conversely, the functions of the ATPase, which eventually include DNA cleavage, evolve independently of selection for triggering of shell transitions. An intermediate result is a non-classical, packaging ATPase/connector-dependent cycle that resembles the type 2 cycle of Figure 5. I proposed a type 2 cycle of this type in [46] before the data definitively demonstrated the existence of a power stroke.

The final result is the evolution of the ATPase/connector-, power stroke-dependent, classical type 1 cycle discussed above. The type 1 cycle displaces the type 2 cycle at the beginning of packaging and possibly continues without intercession of the type 2 cycle when packaging occurs without the high concentration of non-DNA molecules that are in a bacterial cell (for example, in some *in vitro* systems). However, this further evolution does not eliminate the type 2 cycle because selective pressure for the packaging-associated removal of non-DNA molecules remains. Thus, the hypothesis concludes by proposing that the type 2 cycle of classical DNA packaging motors is activated whenever the type 1 cycle both stalls and does not re-start by itself, as discussed above.

In relation to the cycle of Figures 5a–f, h, the selective advantages of evolving the connector/packaging ATPase-dependent type 1 cycle include (1) even tighter packing of DNA and, therefore, more information storage per volume and (2) increased speed of packaging. Similarly, ATPases provide major, although usually different, functions in the power stroke-dependent cycles of all studied biotic motors (reviews: [1–8]). These cycles appear complex enough that they could not have evolved unless they had simpler ancestors, including a thermal ratchet-like ancestor.

Perhaps, this thermal ratchet-like ancestor is a type 2 cycle of a DNA packaging motor. In this case, both prokaryotic and eukaryotic, power stroke-based cellular motors branch after evolution of the type 1 cycle. A possible similarity of one proposed type 1 DNA packaging motor to ABC transporters has previously been noted [18]. That is to say, perhaps an ancient DNA packaging ATPase is the original “transporting” ATPase ancestor. If so, then the DNA packaging ATPase is an ancestor of the RNA packaging ATPase of *Cystoviridae*. This latter sequence of ancestors is, of course, the opposite of the sequence that would be assumed if one considered only the widely accepted conjecture that the RNA world occurred before DNA was a source of encoded information.

The reasoning-from-history strategy used here also produces the proposal that classical DNA packaging motors have the following imprints from the pre-classical motors described in this and the previous section. (1) An observed hyper-expansion of the shells of T3 DNA packaging intermediates [45] is an imprint of, first, abiotic shells in Figures 2 and 3 and, then, the adapted version of this imprint in the pre-classically-derived thermal ratchet of Figure 5. (2) A proposed (not demonstrated [18]) force-transducing role of the connector in the type 1 cycle is an imprint of pre-classical cycles that, first, had the connector acting as a DNA binding/releasing agent (Figures 5a–f, h) and then had the connector losing this role to the DNA packaging ATPase. These previously proposed aspects of DNA packaging are relatively hard to accept when ancestry and evolution are not considered, but are far more acceptable when considered in the context of ancestry.

## 5. Related Points

### 5.1. Location of the Proposed Abiotic Shells

Although not an essential aspect of the hypothesis presented here, the location of abiotic ancestors is a peripheral aspect for which some information does exist. Several lines of reasoning have led to the proposal that abiotic ancestors of current biotic systems are located either below the surface of the solid portions of the earth or at the ocean floor. The formations generated by hydrothermal vents are one specific possibility. The lines of reasoning include the difficulty in imagining anywhere else where both high concentrations of abiotic precursors and adequate chemical potential gradients exist (reviews: [40,55–57,76]).

The possibility of deep earth ancestors raises the additional possibility that these ancestors still exist, if not inside the earth, then perhaps inside other planets where competition has not yet arisen. I speculate that the proposed abiotic shells have surfaces that are positively charged because they eventually bind negatively charged RNA molecules. I note that the basic procedures for detecting and characterizing biotic shells ([45], for example) are also potentially useful for abiotic shells.

The details of the geologically produced motion proposed for the shells in Figures 2 and 3 are potentially embedded in the possibility that, in contrast to the original assertion of a “hot” origin of the earth, the earth is the product of a process cold enough so that trapping of gas (mostly methane) is sufficient to have caused cyclical movements within both crust and mantle [54,55,120,121]. Thus, I speculate that the movement of abiotic shells in Figure 2 occurred below the earth’s surface. Interestingly, synthesis of the rare earth borides requires high temperatures (∼2000 °K), a temperature achieved in the earth’s mantle [61].

### 5.2. Nature and Tests of the Hypothesis

The reasoning used here is different from the reductionist reasoning usually used. Reductionist reasoning starts with chemically defined reactions that, in the laboratory, produce biotic molecules via a pathway that might mimic the pathway historically used. However, reductionist reasoning, which has produced much interesting chemistry, has not yielded the historically used pathway (recent reviews: [40,56,122,123]). The reasoning used in producing the hypothesis above might be called reverse reductionist because the start is the final product and the end is a hypothesis that specifies reactions that are not, in general, chemically defined. The details of these reactions will be learned while performing tests of the hypothesis, if the hypothesis is correct.

The first test proposed here is a search for either the proposed abiotic shells or their fossils. The major problems of such tests will be (1) preserving shells, especially if they were formed and exist at high pressure, (2) establishing an ancestor succession, even if the shells are found and have interesting contents. The latter might be possible by radiological dating of the shells, if found, and their contents. Tests of this type have the disadvantage that a negative result is not conclusive because of the possibility that the shells either are lost during the test or no longer exist.

The second test is a search for pre-classical, biotic ancestors. Our recent finding of aggregating RNA viruses [77,78] raises the possibility that some, not-yet-detected viruses retain ancestral forms of viral propagation. Isolating and characterizing these viruses, assuming that they exist, appears to have potential for both testing the ideas presented here and investigating causes of disease states that are not yet completely understood. For example, this enterprise might reveal RNA viruses intermediate in characteristics to aggregating and segmented-genome RNA viruses. In addition, an explanation based on a virus of these unconventional types is possible for diseases that do not yet have an adequate explanation, motor neuron disease, for example. That is to say, improved probing for unconventional viruses appears to have more than a little importance. The ideas presented here can be used as a working scaffold to at least begin this process.

The third test targets the accuracy of the most uncertain assumption made here. This assumption is that an observable phenomenon, the phage DNA packaging motor, retains some characteristics of the earliest ancestors of all living systems (assumption 5). A test of the accuracy of this assumption also appears to be the isolation and characterization of ancestors, in this case living versions of pre-classical double-stranded DNA phages, *i.e.*, phages whose characteristics are less diverged from ancestral phages than those of the classical double-stranded DNA phages. This venture may be possible with current technology for the following reason. The less diverged phages will have a DNA packing density lower than that of the classical phages, which, thus far, are almost identical in DNA packaging density (about half of the volume occupied by DNA) [75,116].

Because the hydration of unpackaged DNA is relatively large (∼3 g water/gm DNA) in media with relatively low water activity, the hydration of packaged phage DNA increases as the DNA packing density decreases in these media [75]. Based on these principles, buoyant density centrifugation media do exist for selectively isolating phages that have relatively low DNA packaging densities [75].

## 6. Components of the Hypothesis

The following is a summary of the components of the overall hypothesis presented here.

### 6.1. At the Abiotic Origin

The synthesis of precursors of biotic polymers begins with transport-dependent development of chemical potential gradients. The transport occurs in abiotic shells that both package and protect the precursors. These shells move via geologically derived forces.

### 6.2. Also at the Abiotic Origin

Some shells have an expansion/contraction cycle that makes molecule packaging and, therefore, transport-driven synthesis more efficient. The expansion/contraction cycle is driven by binding/releasing of a high-energy compound, possibly methane hydrate. Binding causes the expansion, possibly via cooperatively increased hydration of the shell-forming atoms. An imprint of the characteristics of these shells is transmitted to the viral capsids of today.

### 6.3. Just After the Abiotic Origin

The transport-driven synthesis acquires autocatalytic character when the products of this synthesis stimulate post-transport release of their precursors from shells. This process advances when products become part of the shell and dimerization of the products stimulates release of the precursors.

### 6.4. On the Way to Enzymes and a Complementary Pair of Polymers

The dimerizing products acquire palindromic character, which is the foundation for the acquiring of both enzymatic activity and complementary pairing of single chains. The introduction of palindromic character is potentially the missing link that resolves a previously presented paradox of feasibility [124] for the production, via accidents of chemistry, of complementary polymer chains that replicate genes.

### 6.5. On the Way to RNA Cells

The reactions of transported molecules occur in the interior of the transporting shells, which now transport not only the original precursors, but also products. These reactions become enzymatic in character, as the palindromic character of some of the products introduces the complexity of structure needed for enzymatic activity. The first templated replication occurs.

### 6.6. A Jump to RNA Cells

After a gap in the sequence of proposed ancestors, abiotic shells with associated RNA become RNA cells and viruses. Aggregation-derived redundancy is the initial foundation for bypassing limitations caused by the inaccuracy of RNA-based information transfer. Post-aggregation fusion generates a protein synthesizing system and additional complex biochemistry. Fusion is also the source of the progressive differentiation of viruses from cells. A virus for any given cell is a shell (with shell-integrated RNA) that (a) has a copy of one of the shell-associated RNAs that previously was part of a shell that fused to form the cell to be infected, and (b) therefore recognizes the cell via complementary base pairing of RNAs. Generation of complexity via fusion is a version of horizontal transfer, previously proposed to be a means for early evolution [125]. In the current hypothesis, this stage does not have a significant contribution from fission. In contrast, fission, together with fusion, has previously been proposed to be essential for the production of the first viruses at a later (biotic) stage [126].

### 6.7. A Jump to a Biotic Cell Envelope

The hypothesis leaves another gap at the formation of the first biotic cell membranes and cell walls. Possible chemistries involved have been previously discussed [80].

### 6.8. DNA Viruses and Thermal Ratchet-Based DNA Packaging

After emergence of DNA genomes, double-stranded DNA viruses initially package a genome via shell expansion that is an imprint from the abiotic shells. Single-stranded nucleic acid viruses evolve away from this imprint because of the capacity for nucleic acid/protein co-evolution introduced by palindrome-based structures of single-stranded nucleic acids. The shell expansion-driven packaging of double-stranded DNA evolves to acquire thermal ratchet character, via selection for increase in both the speed of DNA packaging and the amount of DNA packaged. The thermal ratchet includes a connector that provides a DNA bind/release cycle. The bind/release cycle is interleaved with a shell expansion/contraction cycle that results in the packaging of a DNA genome, coupled with the expulsion of non-DNA molecules that had been accidentally packaged.

### 6.9. A DNA Packaging ATPase-Based DNA Packaging Motor

The thermal ratchet evolves a DNA packaging ATPase, via selection for the increased efficiency of dividing functions between two proteins. The DNA packaging ATPase subsequently evolves a power stroke via selection for the increased speed and force of a power stroke-based (type 1) cycle. But, the thermal ratchet-based cycle is retained as a “back-up” (type 2) cycle because of selection for its capacity to increase the density of packaged DNA by expelling accidentally packaged non-DNA molecules.

## References

[1] Alberts B, Miake-Lye R (1992). Unscrambling the puzzle of biological machines: The importance of the details. Cell.

[2] Córdova NJ, Ermentrout B, Oster GF (1992). Dynamics of single-motor molecules: The thermal ratchet model. Proc Natl Acad Sci U S A.

[3] Ishii Y, Taniguchi Y, Iwaki M, Yanagida T (2008). Thermal fluctuations biased for directional motion in molecular motors. BioSystems.

[4] Mickler M, Schleiff E, Hügel T (2008). From biological towards artificial molecular motors. Chemphyschem.

[5] Howard J (2009). Motor proteins as nanomachines: The roles of thermal fluctuations in generating force and motion. Séminaire Poincaré.

[6] Iwaki M, Iwane AH, Shimokawa T, Cooke R, Yanagida T (2009). Brownian search-and-catch mechanism for myosin-VI steps. Nat Chem Biol.

[7] Hänggi P, Marchesoni F (2009). Artificial Brownian motors: Controlling transport on the nanoscale. Rev Mod Phys.

[8] Spirin AS (2009). The Ribosome as a conveying thermal ratchet machine. J Biol Chem.

[9] Aathavan K, Politzer AT, Kaplan A, Moffitt JR, Chemla YR, Grimes S, Jardine PJ, Anderson DL, Bustamante C (2009). Substrate interactions and promiscuity in a viral DNA packaging motor. Nature.

[10] Simpson AA, Tao Y, Leiman PG, Badasso MO, He Y, Jardine PJ, Olson NH, Morais MC, Grimes S, Anderson DL (2000). Structure of the bacteriophage phi29 DNA packaging motor. Nature.

[11] Rao VB, Feiss M (2008). The bacteriophage DNA packaging motor. Annu Rev Genet.

[12] Lee TJ, Schwartz C, Guo P (2009). Construction of bacteriophage phi29 DNA packaging motor and its applications in nanotechnology and therapy. Ann Biomed Eng.

[13] Sun S, Kondabagil K, Draper B, Alam TI, Bowman VD, Zhang Z, Hegde S, Fokine A, Rossmann MG, Rao VB (2008). The structure of the phage T4 DNA packaging motor suggests a mechanism dependent on electrostatic forces. Cell.

[14] Yu J, Moffitt J, Hetherington CL, Bustamante C, Oster G (2010). Mechanochemistry of a viral DNA packaging motor. J Mol Biol.

[15] Ray K, Sabanayagam CR, Lakowicz JR, Black LW (2010). DNA crunching by a viral packaging motor: Compression of a procapsid-portal stalled Y-DNA substrate. Virology.

[16] Lebedev AA, Krause MH, Isidro AL, Vagin AA, Orlova EV, Turner J, Dodson EJ, Tavares P, Antson AA (2007). Structural framework for DNA translocation via the viral portal protein. EMBO J.

[17] Oliveira L, Cuervo A, Tavares P (2010). Direct interaction of the bacteriophage SPP1 packaging ATPase with the portal protein. J Biol Chem.

[18] Serwer P (2010). A Hypothesis for Bacteriophage DNA Packaging Motors. Viruses.

[19] Higgins CF (2007). Multiple molecular mechanisms for multidrug resistance transporters. Nature.

[20] Linton KJ (2007). Structure and function of ABC transporters. Physiology.

[21] Gutmann DA, Ward A, Urbatsch IL, Chang G, van Veen HW (2010). Understanding polyspecificity of multidrug ABC transporters: Closing in on the gaps in ABCB1. Trends Biochem Sci.

[22] Kurakin A (2007). Self-organization *versus* watchmaker: Ambiguity of molecular recognition and design charts of cellular circuitry. J Mol Recognit.

[23] Kurakin A (2010). Order without design. Theor Biol Med Modell.

[24] Sheaffer AK, Newcomb WW, Gao M, Yu D, Weller SK, Brown JC, Tenney DJ (2001). Herpes simplex virus DNA cleavage and packaging proteins associate with the procapsid prior to its maturation. J Virol.

[25] Baker ML, Jiang W, Rixon FJ, Chiu W (2005). Common ancestry of herpesviruses and tailed DNA bacteriophages. J Virol.

[26] Johnson JE, Chiu W (2007). DNA packaging and delivery machines in tailed bacteriophages. Curr Opin Struct Biol.

[27] Krupovic M, Bamford D (2008). Virus evolution: How far does the double β-barrel viral lineage extend. Nat Rev.

[28] Koonin EV, Yutin N (2009). Origin and evolution of eukaryotic large nucleo-cytoplasmic DNA viruses. Intervirology.

[29] Jiang W, Baker ML, Jakana J, Weigele PR, King J, Chiu W (2008). Backbone structure of the infectious epsilon 15 virus capsid revealed by electron cryomicroscopy. Nature.

[30] Black LW (1995). DNA packaging and cutting by phage terminases: Control in phage T4 by a synaptic mechanism. Bioessays.

[31] Sheaffer AK, Newcomb WW, Gao M, Yu D, Weller SK, Brown JC, Tenney DJ (2001). Herpes simplex virus DNA cleavage and packaging proteins associate with the procapsid prior to its maturation. J Virol.

[32] Serwer P, Hayes SJ, Zaman S, Lieman K, Rolando M, Hardies SC (2004). Improved isolation of undersampled bacteriophages: Finding of distant terminase genes. Virology.

[33] Iyer LM, Makarova KS, Koonin EV, Aravind L (2004). Comparative genomics of the FtsK-HerA superfamily of pumping ATPases: Implications for the origins of chromosome segregation, cell division and viral capsid packaging. Nucleic Acids Res.

[34] Ponchon L, Boulanger P, Labesse G, Letellier L (2006). The endonuclease domain of bacteriophage terminases belongs to the resolvase/integrase/ribonuclease H superfamily: A bioinformatics analysis validated by a functional study on bacteriophage T5. J Biol Chem.

[35] Burroughs AM, Iyer LM, Aravind L (2007). Comparative genomics and evolutionary trajectories of viral ATP dependent DNA-packaging systems. Genome Dyn.

[36] Agirrezabala X, Martin-Benito J, Valle M, Gonzalez JM, Valencia AJ, Valpuesta JM, Carrascosa JL (2005). Structure of the connector of bacteriophage T7 at 8Å resolution: Structural homologies of a basic component of a DNA translocating machinery. J Mol Biol.

[37] Newcomb WW, Juhas RM, Thomsen DR, Homa FL, Burch AD, Weller SK, Brown JC (2001). The UL6 gene product forms the portal for entry of DNA into the herpes simplex virus capsid. J Virol.

[38] Newcomb WW, Cockrell SK, Homa FL, Brown JC (2009). Polarized DNA injection from the herpesvirus capsid. J Mol Biol.

[39] Bamford DH, Burnett RM, Stuart DI (2002). Evolution of viral structure. Theor Popul Biol.

[40] Martin W, Russell MJ (2003). On the origins of cells: A hypothesis for the evolutionary transitions from abiotic geochemistry to chemoautotrophic prokaryotes, and from prokaryotes to nucleated cells. Philos Trans R Soc Lond B.

[41] Kutschera U, Niklas KJ (2004). The modern theory of biological evolution: An expanded synthesis. Naturwissenschaften.

[42] Catalano CE (2000). The terminase enzyme from bacteriophage lambda: A DNA-packaging machine. Cell Mol Life Sci.

[43] Petrov AS, Harvey SC (2008). Packaging double-helical DNA into viral capsids: Structures, forces, and energetics. Biophys J.

[44] Sun S, Rao VB, Rossmann MG (2010). Genome packaging in viruses. Curr Opin Struct Biol.

[45] Serwer P, Wright ET, Hakala K, Weintraub ST, Su M, Jiang W (2010). DNA packaging-associated hyper-capsid expansion of bacteriophage T3. J Mol Biol.

[46] Serwer P (2003). Models of bacteriophage DNA packaging motors. J Struct Biol.

[47] Smith DE, Tans SJ, Smith SB, Grimes S, Anderson DL, Bustamante C (2001). The bacteriophage straight phi29 portal motor can package DNA against a large internal force. Nature.

[48] Oram M, Sabanayagam C, Black LW (2008). Modulation of the packaging reaction of bacteriophage t4 terminase by DNA structure. J Mol Biol.

[49] Mindich L (2004). Packaging, replication and recombination of the segmented genomes of bacteriophage □6 and its relatives. Virus Res.

[50] Kainov DE, Tuma R, Mancini EJ (2006). Hexameric molecular motors: P4 packaging ATPase unravels the mechanism. Cell Mol Life Sci.

[51] Butcher SJ, Dokland T, Ojala PM, Bamford DH, Fuller SD (1997). Intermediates in the assembly pathway of the double-stranded RNA virus phi6. EMBO J.

[52] Karhu NJ, Ziedaite G, Bamford DH, Bamford JKH (2007). Efficient DNA packaging of bacteriophage PRD1 requires the unique vertex protein P6. J Virol.

[53] Butcher SJ, Bamford DH, Fuller SD (1995). DNA packaging orders the membrane of bacteriophage PRD1. EMBO J.

[54] Gold T (1992). The deep, hot biosphere. Proc Natl Acad Sci U S A.

[55] Gold T (1999). The Deep Hot Biosphere.

[56] Koonin EV (2009). On the origin of cells and viruses: Primordial virus world scenario. Ann N Y Acad Sci.

[57] Nitschke W, Russell MJ (2009). Hydrothermal focusing of chemical and chemiosmotic energy, supported by delivery of catalytic Fe, Ni, Mo/W, Co, S and Se, forced life to emerge. J Mol Evol.

[58] Vetsigian K, Woese C, Goldenfeld N (2006). Collective evolution and the genetic code. Proc Natl Acad Sci U S A.

[59] Woese CR, Goldenfeld N (2009). How the microbial world saved evolution from the scylla of molecular biology and the charybdis of the modern synthesis. Microbiol Mol Biol Rev.

[60] Van der Put PJ (2001). The Inorganic Chemistry of Materials: How to Make Things out of Elements.

[61] Mori T (2009). Novel physical properties of rare earth higher borides. J Phys Conf Ser.

[62] Pauling L, Marsh RE (1952). The structure of chlorine hydrate. Proc Natl Acad Sci U S A.

[63] Sloan ED (1998). Clathrate Hydrates of Natural Gases.

[64] Zhang J, Hawtin RW, Yang Y, Nakagava E, Rivero M, Choi SK, Rodger PM (2008). Molecular dynamics study of methane hydrate formation at a water/methane interface. J Phys Chem B.

[65] Pauling L (1970). General Chemistry.

[66] Tielrooij KJ, Garcia-Araez N, Bonn M, Bakker HJ (2010). Cooperativity in ion hydration. Science.

[67] Glasby GP (2006). Abiogenic origin of hydrocarbons: An historical overview. Resour Geol.

[68] Greenwood NN, Earnshaw A (1984). Chemistry of the Elements.

[69] Kauffman S (1995). At Home in the Universe: The Search for the Laws of Self-Organization and Complexity.

[70] Vlassov AV, Kazakov SA, Johnston BH, Landweber LF (2005). The RNA world on ice: A new scenario for the emergence of RNA information. J Mol Evol.

[71] Forterre P (2005). The two ages of the RNA world, and the transition to the DNA world: A story of viruses and cells. Biochemie.

[72] Goldenfeld N, Woese C (2007). Biology’s next revolution. Nature.

[73] Cech TR (2009). Evolution of biological catalysis: Ribozyme to RNA enzyme. Cold Spring Harbor Symp Quant Biol.

[74] Pace NR (1991). Origin of life—Facing up to the physical setting. Cell.

[75] Serwer P (1975). Buoyant density sedimentation of macromolecules in sodium iothalamate density gradients. J Mol Biol.

[76] Martin W, Baross J, Kelley D, Russell MJ (2008). Hydrothermal vents and the origin of life. Nat Rev Microbiol.

[77] Serwer P, Hayes SJ, Thomas JA, Demeler B, Hardies SC (2009). Isolation of novel large and aggregating bacteriophages. Methods Mol Biol.

[78] Serwer P, Wang H (2005). Single-particle light microscopy of bacteriophages. J Nanosci Nanotechnol.

[79] Pais-Correia A-M, Sachse M, Guadagnini S, Robbiati V, Lasserre R, Gessain A, Gout O, Alcover A, Thoulouze M-I (2010). Biofilm-like extracellular viral assemblies mediate HTLV-1 cell-to-cell transmission at virological synapses. Nat Med.

[80] Wächtershäuser G (2007). On the chemistry and evolution of the pioneer organism. Chem Biodiversity.

[81] Serwer P (2007). Evolution and the complexity of bacteriophages. Virol J.

[82] Moreau H, Piganeau G, Desdevises Y, Cooke R, Derelle E, Grimsley N (2010). Marine prasinovirus genomes show low evolutionary divergence and acquisition of protein metabolism genes by horizontal gene transfer. J Virol.

[83] Filee J, Chandler M (2010). Gene exchange and the origin of giant viruses. Intervirology.

[84] Liu H, Fu Y, Jiang D, Li G, Xie J, Cheng J, Peng Y, Ghabrial SA, Yi X (2010). Widespread horizontal gene transfer from double-stranded RNA viruses to eukaryotic nuclear genomes. J Virol.

[85] Baker TS, Newcomb WW, Olson NH, Cowsert LM, Olson C, Brown JC (1991). Structures of bovine and human papillomaviruses. Analysis by cryoelectron microscopy and three-dimensional image reconstruction. Biophys J.

[86] Belnap DM, Olson NH, Cladel NM, Newcomb WW, Brown JC, Kreider JW, Christensen ND, Baker TS (1996). Conserved features in papillomavirus and polyomavirus capsids. J Mol Biol.

[87] Ben-nun-Shaul O, Bronfeld H, Reshef D, Schueler-Furman O, Oppenheim A (2009). The SV40 capsid is stabilized by a conserved pentapeptide hinge of the major capsid protein VP1. J Mol Biol.

[88] Prangishvili D, Forterre P, Garrett RA (2006). Viruses of the archaea: A unifying view. Nat Rev Microbiol.

[89] Bize A, Peng X, Prokofeva M, Maclellan K, Lucas S, Forterre P, Garrett RA, Bonch-Osmolovskaya EA, Prangishvili D (2008). Viruses in acidic geothermal environments of the Kamchatka Peninsula. Res Microbiol.

[90] Prangishvili D, Garrett RA, Koonin EV (2006). Evolutionary genomics of archaeal viruses: Unique viral genomes in the third domain of life. Virus Res.

[91] Huet A, Conway JF, Letellier L, Boulanger P (2010). *In vitro* assembly of the T = 13 procapsid of bacteriophage T5 with its scaffolding domain. J Virol.

[92] Dokland T (1999). Scaffolding proteins and their role in viral assembly. Cell Mol Life Sci.

[93] Johnson JE (2008). Multi-disciplinary studies of viruses: The role of structure in shaping the questions and answers. J Struct Biol.

[94] Fane BA, Prevelige PE (2003). Mechanism of scaffolding-assisted viral assembly. Adv Protein Chem.

[95] Bândea CI (1983). A new theory on the origin and the nature of viruses. J Theor Biol.

[96] Forterre P (2010). Defining life: The virus viewpoint. Origins Life Evol Biospheres.

[97] Summers WC (1999). Félix d’Herelle and the Origins of Molecular Biology.

[98] D’Herelle F (1933). The Bacteriophage and Its Clinical Application.

[99] Forterre P Three RNA cells for ribosomal lineages and three DNA viruses to replicate their genomes: A hypothesis for the origin of cellular domain. Proc Natl Acad Sci U S A.

[100] Cue D, Feiss M (1997). Genetic evidence that recognition of cosQ, the signal for termination of phage lambda DNA packaging, depends on the extent of head filling. Genetics.

[101] Catalano CE (2000). The terminase enzyme from bacteriophage lambda: A DNA-packaging machine. Cell Mol Life Sci.

[102] Maluf NK, Gaussier H, Bogner E, Feiss M, Catalano CE (2006). Assembly of bacteriophage lambda terminase into a viral DNA maturation and packaging machine. Biochemistry.

[103] Hashimoto C, Fujisawa H (1988). Packaging and transduction of non-T3 DNA by bacteriophage T3. Virology.

[104] Son M, Serwer P (1992). Role of exonuclease in the specificity of bacteriophage T7 DNA packaging. Virology.

[105] Serwer P, Watson RH, Hayes SJ (1982). Detection and characterization of agarose-binding, capsid-like particles produced during assembly of a bacteriophage T7 procapsid. J Virol.

[106] Serwer P, Watson RH (1982). Function of an internal bacteriophage T7 core during assembly of a T7 procapsid. J Virol.

[107] Bazinet C, King J (1988). Initiation of P22 procapsid assembly *in vivo*. J Mol Biol.

[108] Dokland T (2000). Freedom and restraint: Themes in virus capsid assembly. Structure.

[109] Johnson JE (2010). Virus particle maturation: Insights into elegantly programmed nanomachines. Curr Opin Struct Biol.

[110] Katen S, Zlotnick A (2009). The thermodynamics of virus capsid assembly. Methods Enzymol.

[111] Ackermann H-W, Kropinski AM (2007). Curated list of prokaryote viruses with fully sequenced genomes. Res Microbiol.

[112] Cherwa JE, Fane BA (2009). Complete virion assembly with scaffolding proteins altered in the ability to perform a critical conformational switch. J Virol.

[113] Aksyuk AA, Rossmann MG (2011). Bacteriophage assembly. Viruses.

[114] Uchiyama A, Fane BA (2005). Identification of an interacting coat-external scaffolding protein domain required for both the initiation of phiX174 procapsid morphogenesis and the completion of DNA packaging. J Virol.

[115] Strauss SK, Scott WR, Symmons MF, Marvin DA (2008). On the structures of filamentous bacteriophage Ff (fd, f1, M13). Eur Biophys J.

[116] Forrey C, Muthukumar M (2006). Langevin dynamics simulations of genome packing in bacteriophage. Biophys J.

[117] Casjens S, Wyckoff E, Hayden M, Sampson L, Eppler K, Randall S, Moreno ET, Serwer P (1992). Bacteriophage P22 portal protein is part of the gauge that regulates packing density of intravirion DNA. J Mol Biol.

[118] Isidro A, Henriques AO, Tavares P (2004). The portal protein plays essential roles at different steps of the SPP1 DNA packaging process. Virology.

[119] Zheng H, Olia AS, Gonen M, Andrews S, Cingolani G, Gonen T (2008). A conformational switch in bacteriophage p22 portal protein primes genome injection. Mol Cell.

[120] Kudryavtsev N (1951). Against the organic hypothesis of the origin of petroleum. Pet Econ.

[121] Herndon JM (2006). Enhanced prognosis for abiotic natural gas and petroleum resources. Curr Sci.

[122] Orgel LE (2004). Prebiotic chemistry and the origin of the RNA world. Crit Rev Biochem Mol Biol.

[123] Orgel LE (2008). The implausibility of metabolic cycles on the prebiotic Earth. PLoS Biol.

[124] De Duve C (2007). Chemistry and selection. Chem Biodiversity.

[125] Woese CR (2002). On the evolution of cells. Proc Natl Acad Sci U S A.

[126] Bândea CI (2009). A unifying scenario on the origin and evolution of cellular and viral domains. Nature Precedings.

